# Editorial note

**DOI:** 10.1098/rsta.2023.0172

**Published:** 2023-10-02

**Authors:** Editorial office

This issue is the second part of a two-part issue on ‘Thermodynamics 2.0: Bridging the natural and social sciences’. Part 1 can be found at https://royalsocietypublishing.org/toc/rsta/2023/381/2252. The Preface to Part 1 contains a brief summary of the topic for both issues [1].

**Conflict of interest declaration**

This theme issue was put together by the Guest Editor team under supervision from the journal's Editorial staff, following the Royal Society's ethical codes and best-practice guidelines. The Guest Editor team invited contributions and handled the review process. Individual Guest Editors were not involved in assessing papers where they had a personal, professional or financial conflict of interest with the authors or the research described. Independent reviewers assessed all papers. Invitation to contribute did not guarantee inclusion.
